# Supplementary material for: Shape2SAS – a web application to simulate small-angle scattering data and pair distance distributions from user-defined shapes
Source: ArXiv. 2023 Jan 12:arXiv:2301.04976v1. Preprint. [Version 1] (PMC9882588)
Supplement: 1 [file NIHPP2301.04976V1-supplement-1.pdf]

Supplementary figures

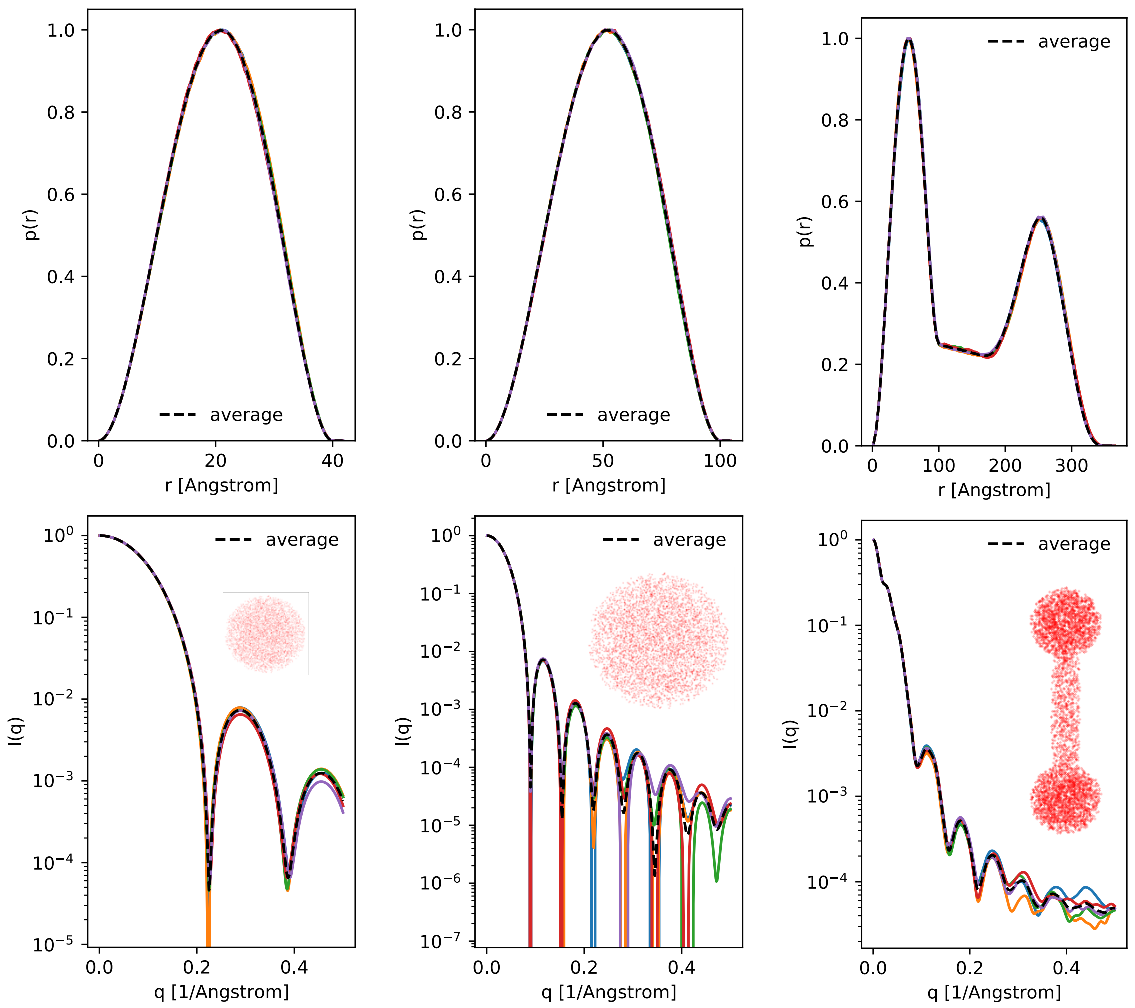

Figure S1. Precision of Shape2SAS when using 5000 points. Calculated pair distance distribution and intensity for five repeated simulations. (a) Sphere with radius 20 Å (b) Sphere with radius 50 Å. (c) Dumbbell composed of a cylinder with radius 50 Å and length 200 Å, and two spheres of radius 20 Å, with their center of mass shifted ±125 Å.
